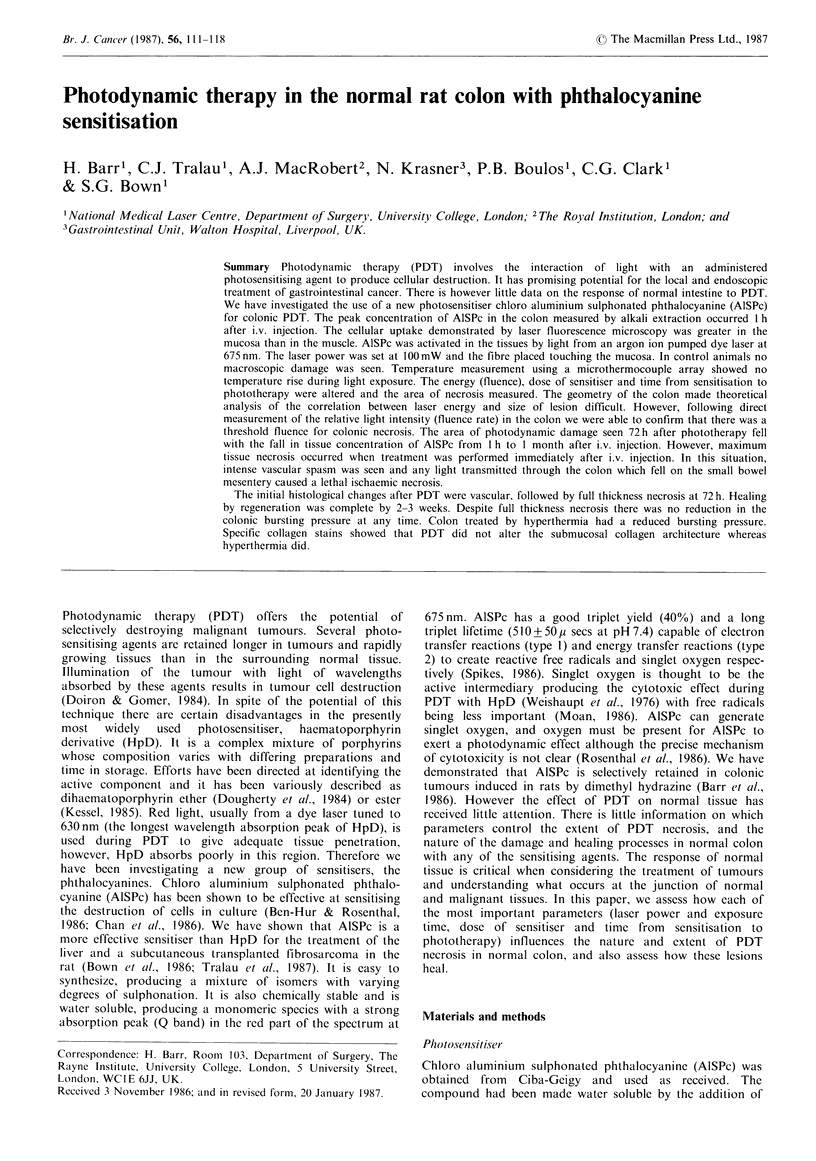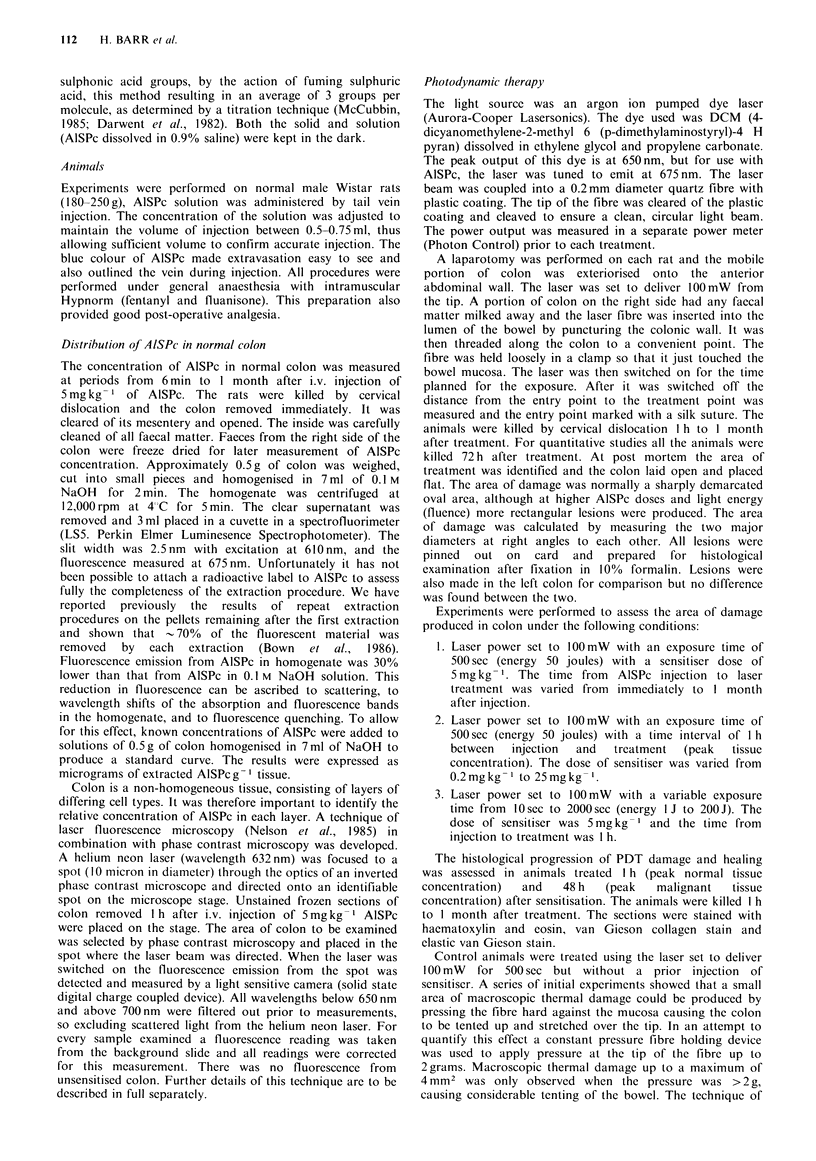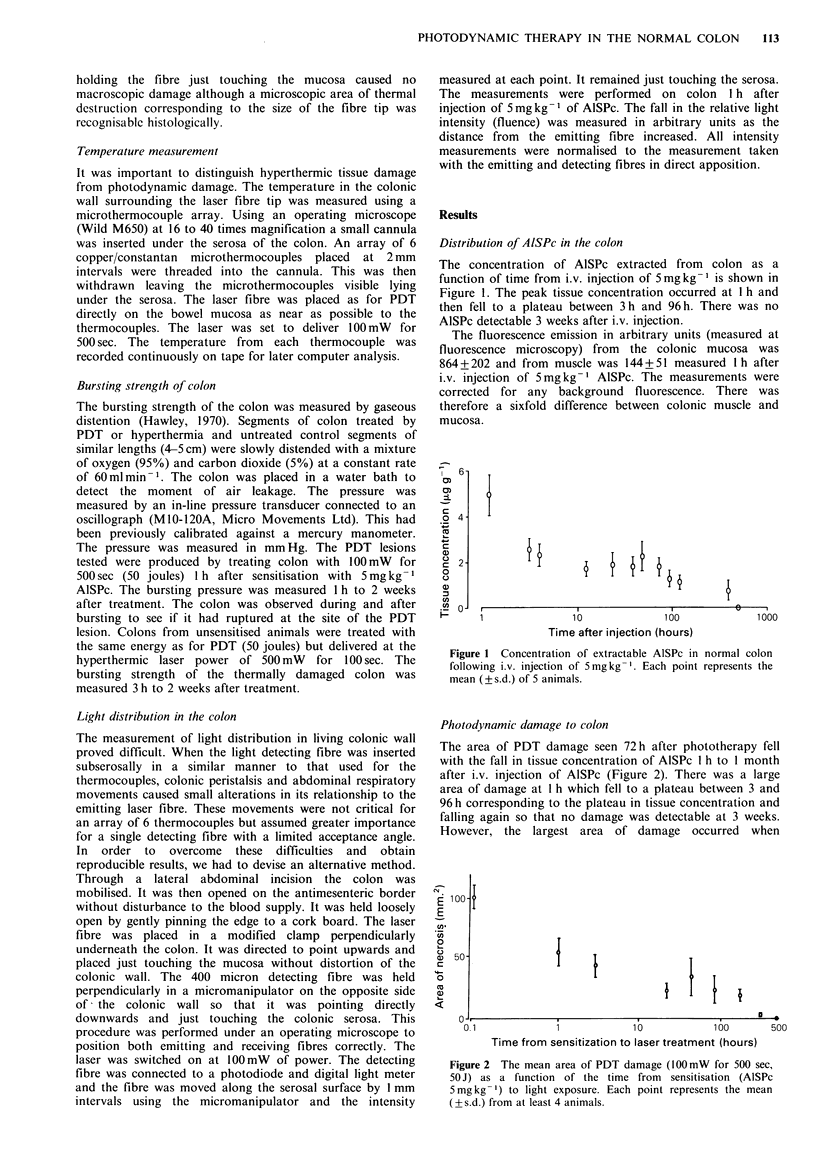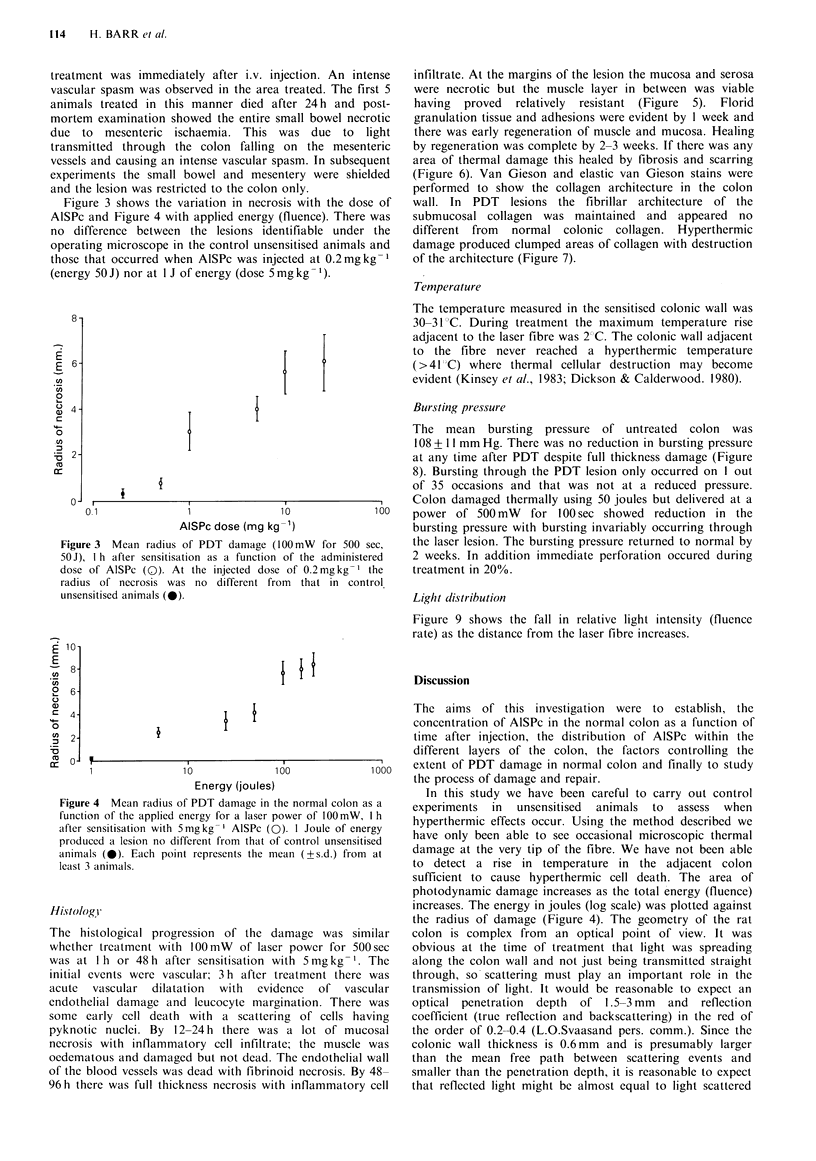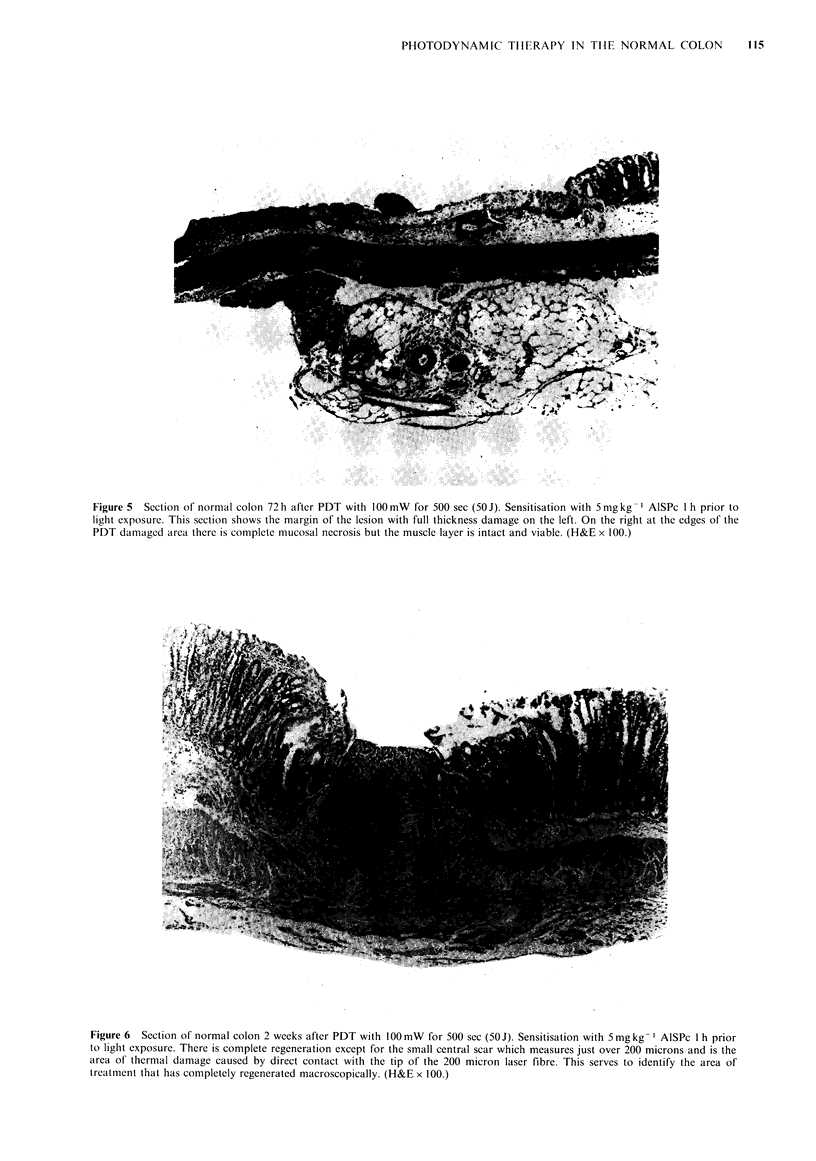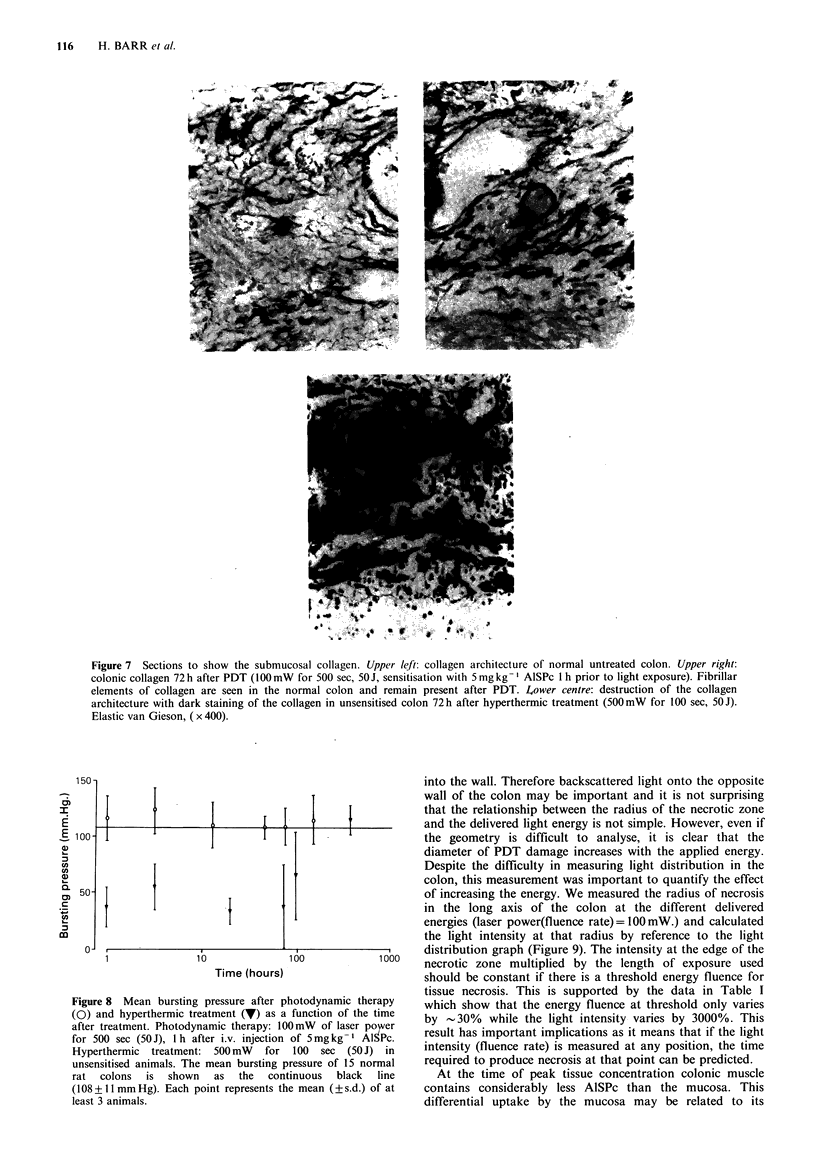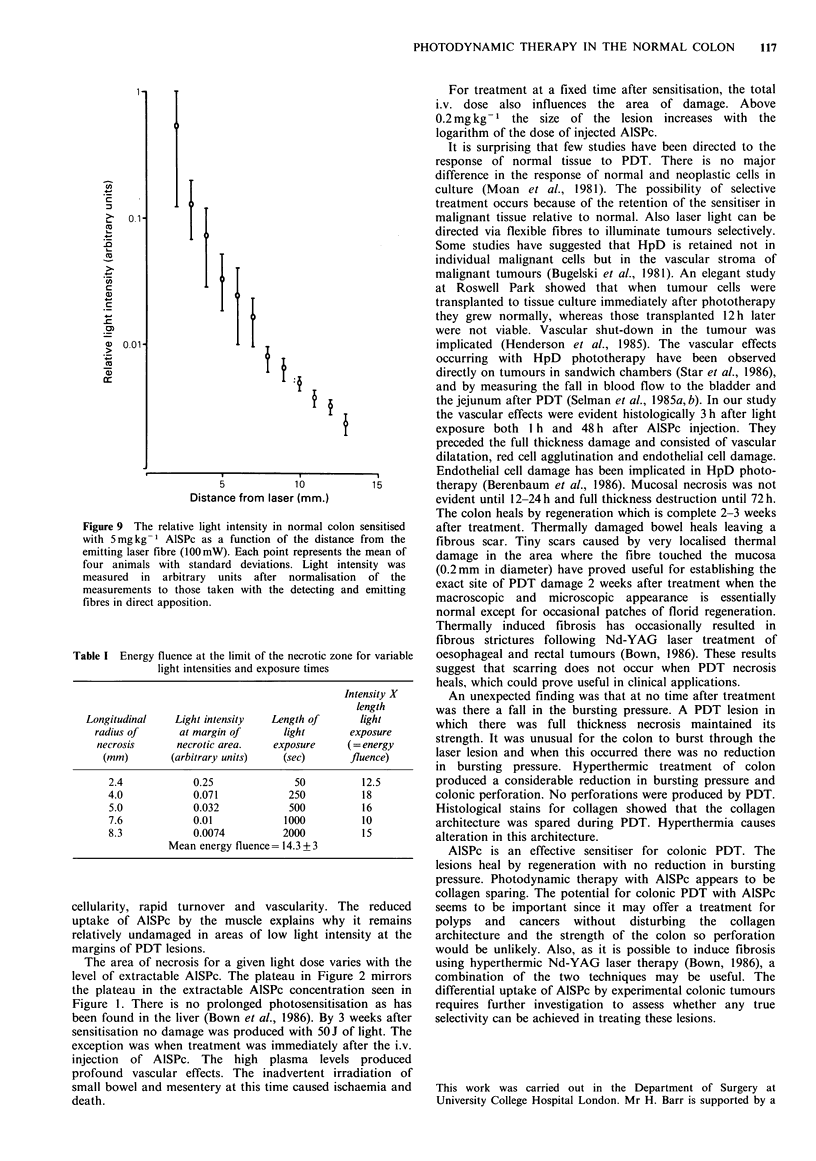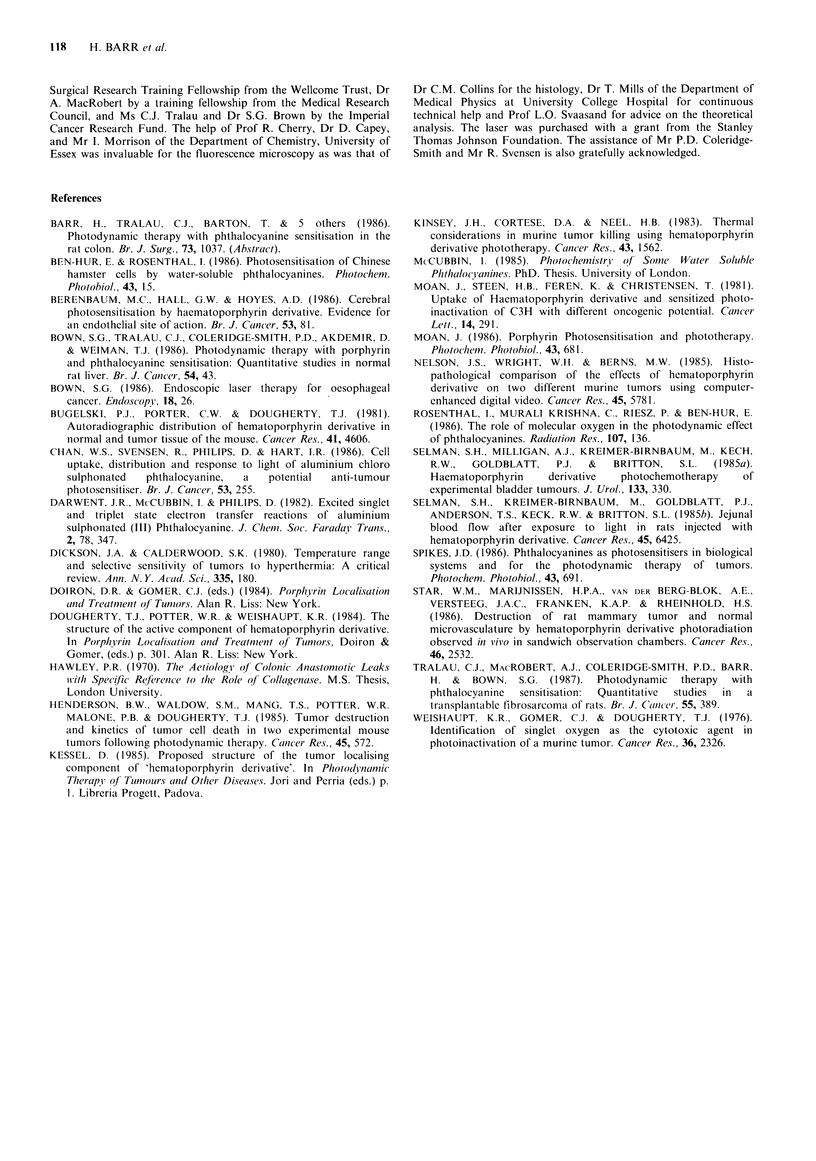# Photodynamic therapy in the normal rat colon with phthalocyanine sensitisation.

**DOI:** 10.1038/bjc.1987.166

**Published:** 1987-08

**Authors:** H. Barr, C. J. Tralau, A. J. MacRobert, N. Krasner, P. B. Boulos, C. G. Clark, S. G. Bown

**Affiliations:** National Medical Laser Centre, Department of Surgery, University College, London, UK.

## Abstract

**Images:**


Br. J. Cancer (1987), 56, 111-118 © The Macmillan Press Ltd., 1987

### Photodynamic therapy in the normal rat colon with phthalocyanine sensitisation

H. Barr[1], C.J. Tralau[1], A.J. MacRobert[2], N. Krasner[3], P.B. Boulos[1], C.G. Clark[1] & S.G. Bown[1]

[1]*National Medical Laser Centre, Department of Surgery, University College, London;* [2]*The Royal Institution, London; and* [3]*Gastrointestinal Unit, Walton Hospital, Liverpool, UK.*

**Summary** Photodynamic therapy (PDT) involves the interaction of light with an administered photosensitising agent to produce cellular destruction. It has promising potential for the local and endoscopic treatment of gastrointestinal cancer. There is however little data on the response of normal intestine to PDT. We have investigated the use of a new photosensitiser chloro aluminium sulphonated phthalocyanine (AlSPc) for colonic PDT. The peak concentration of AlSPc in the colon measured by alkali extraction occurred 1 h after i.v. injection. The cellular uptake demonstrated by laser fluorescence microscopy was greater in the mucosa than in the muscle. AlSPc was activated in the tissues by light from an argon ion pumped dye laser at 675 nm. The laser power was set at 100 mW and the fibre placed touching the mucosa. In control animals no macroscopic damage was seen. Temperature measurement using a microthermocouple array showed no temperature rise during light exposure. The energy (fluence), dose of sensitiser and time from sensitisation to phototherapy were altered and the area of necrosis measured. The geometry of the colon made theoretical analysis of the correlation between laser energy and size of lesion difficult. However, following direct measurement of the relative light intensity (fluence rate) in the colon we were able to confirm that there was a threshold fluence for colonic necrosis. The area of photodynamic damage seen 72 h after phototherapy fell with the fall in tissue concentration of AlSPc from 1 h to 1 month after i.v. injection. However, maximum tissue necrosis occurred when treatment was performed immediately after i.v. injection. In this situation, intense vascular spasm was seen and any light transmitted through the colon which fell on the small bowel mesentery caused a lethal ischaemic necrosis.

The initial histological changes after PDT were vascular, followed by full thickness necrosis at 72 h. Healing by regeneration was complete by 2–3 weeks. Despite full thickness necrosis there was no reduction in the colonic bursting pressure at any time. Colon treated by hyperthermia had a reduced bursting pressure. Specific collagen stains showed that PDT did not alter the submucosal collagen architecture whereas hyperthermia did.

Photodynamic therapy (PDT) offers the potential of selectively destroying malignant tumours. Several photosensitising agents are retained longer in tumours and rapidly growing tissues than in the surrounding normal tissue. Illumination of the tumour with light of wavelengths absorbed by these agents results in tumour cell destruction (Doiron & Gomer, 1984). In spite of the potential of this technique there are certain disadvantages in the presently most widely used photosensitiser, haematoporphyrin derivative (HpD). It is a complex mixture of porphyrins whose composition varies with differing preparations and time in storage. Efforts have been directed at identifying the active component and it has been variously described as dihaematoporphyrin ether (Dougherty *et al.*, 1984) or ester (Kessel, 1985). Red light, usually from a dye laser tuned to 630 nm (the longest wavelength absorption peak of HpD), is used during PDT to give adequate tissue penetration, however, HpD absorbs poorly in this region. Therefore we have been investigating a new group of sensitisers, the phthalocyanines. Chloro aluminium sulphonated phthalocyanine (AlSPc) has been shown to be effective at sensitising the destruction of cells in culture (Ben-Hur & Rosenthal, 1986; Chan *et al.*, 1986). We have shown that AlSPc is a more effective sensitiser than HpD for the treatment of the liver and a subcutaneous transplanted fibrosarcoma in the rat (Bown *et al.*, 1986; Tralau *et al.*, 1987). It is easy to synthesize, producing a mixture of isomers with varying degrees of sulphonation. It is also chemically stable and is water soluble, producing a monomeric species with a strong absorption peak (Q band) in the red part of the spectrum at 675 nm. AlSPc has a good triplet yield (40%) and a long triplet lifetime ($510 \pm 50\,\mu$ secs at pH 7.4) capable of electron transfer reactions (type 1) and energy transfer reactions (type 2) to create reactive free radicals and singlet oxygen respectively (Spikes, 1986). Singlet oxygen is thought to be the active intermediary producing the cytotoxic effect during PDT with HpD (Weishaupt *et al.*, 1976) with free radicals being less important (Moan, 1986). AlSPc can generate singlet oxygen, and oxygen must be present for AlSPc to exert a photodynamic effect although the precise mechanism of cytotoxicity is not clear (Rosenthal *et al.*, 1986). We have demonstrated that AlSPc is selectively retained in colonic tumours induced in rats by dimethyl hydrazine (Barr *et al.*, 1986). However the effect of PDT on normal tissue has received little attention. There is little information on which parameters control the extent of PDT necrosis, and the nature of the damage and healing processes in normal colon with any of the sensitising agents. The response of normal tissue is critical when considering the treatment of tumours and understanding what occurs at the junction of normal and malignant tissues. In this paper, we assess how each of the most important parameters (laser power and exposure time, dose of sensitiser and time from sensitisation to phototherapy) influences the nature and extent of PDT necrosis in normal colon, and also assess how these lesions heal.

Correspondence: H. Barr, Room 103, Department of Surgery, The Rayne Institute, University College, London, 5 University Street, London, WC1E 6JJ, UK.

Received 3 November 1986; and in revised form, 20 January 1987.

### Materials and methods

### *Photosensitiser*

Chloro aluminium sulphonated phthalocyanine (AlSPc) was obtained from Ciba-Geigy and used as received. The compound had been made water soluble by the addition of sulphonic acid groups, by the action of fuming sulphuric acid, this method resulting in an average of 3 groups per molecule, as determined by a titration technique (McCubbin, 1985; Darwent *et al.*, 1982). Both the solid and solution (AlSPc dissolved in 0.9% saline) were kept in the dark.

*Animals*

Experiments were performed on normal male Wistar rats (180–250 g), AlSPc solution was administered by tail vein injection. The concentration of the solution was adjusted to maintain the volume of injection between 0.5–0.75 ml, thus allowing sufficient volume to confirm accurate injection. The blue colour of AlSPc made extravasation easy to see and also outlined the vein during injection. All procedures were performed under general anaesthesia with intramuscular Hypnorm (fentanyl and fluanisone). This preparation also provided good post-operative analgesia.

*Distribution of AlSPc in normal colon*

The concentration of AlSPc in normal colon was measured at periods from 6 min to 1 month after i.v. injection of 5 mg $kg^{-1}$ of AlSPc. The rats were killed by cervical dislocation and the colon removed immediately. It was cleared of its mesentery and opened. The inside was carefully cleaned of all faecal matter. Faeces from the right side of the colon were freeze dried for later measurement of AlSPc concentration. Approximately 0.5 g of colon was weighed, cut into small pieces and homogenised in 7 ml of 0.1 M NaOH for 2 min. The homogenate was centrifuged at 12,000 rpm at 4°C for 5 min. The clear supernatant was removed and 3 ml placed in a cuvette in a spectrofluorimeter (LS5. Perkin Elmer Luminesence Spectrophotometer). The slit width was 2.5 nm with excitation at 610 nm, and the fluorescence measured at 675 nm. Unfortunately it has not been possible to attach a radioactive label to AlSPc to assess fully the completeness of the extraction procedure. We have reported previously the results of repeat extraction procedures on the pellets remaining after the first extraction and shown that ~70% of the fluorescent material was removed by each extraction (Bown *et al.*, 1986). Fluorescence emission from AlSPc in homogenate was 30% lower than that from AlSPc in 0.1 M NaOH solution. This reduction in fluorescence can be ascribed to scattering, to wavelength shifts of the absorption and fluorescence bands in the homogenate, and to fluorescence quenching. To allow for this effect, known concentrations of AlSPc were added to solutions of 0.5 g of colon homogenised in 7 ml of NaOH to produce a standard curve. The results were expressed as micrograms of extracted AlSPc $g^{-1}$ tissue.

Colon is a non-homogeneous tissue, consisting of layers of differing cell types. It was therefore important to identify the relative concentration of AlSPc in each layer. A technique of laser fluorescence microscopy (Nelson *et al.*, 1985) in combination with phase contrast microscopy was developed. A helium neon laser (wavelength 632 nm) was focused to a spot (10 micron in diameter) through the optics of an inverted phase contrast microscope and directed onto an identifiable spot on the microscope stage. Unstained frozen sections of colon removed 1 h after i.v. injection of 5 mg $kg^{-1}$ AlSPc were placed on the stage. The area of colon to be examined was selected by phase contrast microscopy and placed in the spot where the laser beam was directed. When the laser was switched on the fluorescence emission from the spot was detected and measured by a light sensitive camera (solid state digital charge coupled device). All wavelengths below 650 nm and above 700 nm were filtered out prior to measurements, so excluding scattered light from the helium neon laser. For every sample examined a fluorescence reading was taken from the background slide and all readings were corrected for this measurement. There was no fluorescence from unsensitised colon. Further details of this technique are to be described in full separately.

*Photodynamic therapy*

The light source was an argon ion pumped dye laser (Aurora-Cooper Lasersonics). The dye used was DCM (4-dicyanomethylene-2-methyl 6 (p-dimethylaminostyryl)-4 H pyran) dissolved in ethylene glycol and propylene carbonate. The peak output of this dye is at 650 nm, but for use with AlSPc, the laser was tuned to emit at 675 nm. The laser beam was coupled into a 0.2 mm diameter quartz fibre with plastic coating. The tip of the fibre was cleared of the plastic coating and cleaved to ensure a clean, circular light beam. The power output was measured in a separate power meter (Photon Control) prior to each treatment.

A laparotomy was performed on each rat and the mobile portion of colon was exteriorised onto the anterior abdominal wall. The laser was set to deliver 100 mW from the tip. A portion of colon on the right side had any faecal matter milked away and the laser fibre was inserted into the lumen of the bowel by puncturing the colonic wall. It was then threaded along the colon to a convenient point. The fibre was held loosely in a clamp so that it just touched the bowel mucosa. The laser was then switched on for the time planned for the exposure. After it was switched off the distance from the entry point to the treatment point was measured and the entry point marked with a silk suture. The animals were killed by cervical dislocation 1 h to 1 month after treatment. For quantitative studies all the animals were killed 72 h after treatment. At post mortem the area of treatment was identified and the colon laid open and placed flat. The area of damage was normally a sharply demarcated oval area, although at higher AlSPc doses and light energy (fluence) more rectangular lesions were produced. The area of damage was calculated by measuring the two major diameters at right angles to each other. All lesions were pinned out on card and prepared for histological examination after fixation in 10% formalin. Lesions were also made in the left colon for comparison but no difference was found between the two.

Experiments were performed to assess the area of damage produced in colon under the following conditions:

1. Laser power set to 100 mW with an exposure time of 500 sec (energy 50 joules) with a sensitiser dose of 5 mg $kg^{-1}$. The time from AlSPc injection to laser treatment was varied from immediately to 1 month after injection.
2. Laser power set to 100 mW with an exposure time of 500 sec (energy 50 joules) with a time interval of 1 h between injection and treatment (peak tissue concentration). The dose of sensitiser was varied from 0.2 mg $kg^{-1}$ to 25 mg $kg^{-1}$.
3. Laser power set to 100 mW with a variable exposure time from 10 sec to 2000 sec (energy 1 J to 200 J). The dose of sensitiser was 5 mg $kg^{-1}$ and the time from injection to treatment was 1 h.

The histological progression of PDT damage and healing was assessed in animals treated 1 h (peak normal tissue concentration) and 48 h (peak malignant tissue concentration) after sensitisation. The animals were killed 1 h to 1 month after treatment. The sections were stained with haematoxylin and eosin, van Gieson collagen stain and elastic van Gieson stain.

Control animals were treated using the laser set to deliver 100 mW for 500 sec but without a prior injection of sensitiser. A series of initial experiments showed that a small area of macroscopic thermal damage could be produced by pressing the fibre hard against the mucosa causing the colon to be tented up and stretched over the tip. In an attempt to quantify this effect a constant pressure fibre holding device was used to apply pressure at the tip of the fibre up to 2 grams. Macroscopic thermal damage up to a maximum of 4 $mm^2$ was only observed when the pressure was $>2$ g, causing considerable tenting of the bowel. The technique of holding the fibre just touching the mucosa caused no macroscopic damage although a microscopic area of thermal destruction corresponding to the size of the fibre tip was recognisable histologically.

*Temperature measurement*

It was important to distinguish hyperthermic tissue damage from photodynamic damage. The temperature in the colonic wall surrounding the laser fibre tip was measured using a microthermocouple array. Using an operating microscope (Wild M650) at 16 to 40 times magnification a small cannula was inserted under the serosa of the colon. An array of 6 copper/constantan microthermocouples placed at 2 mm intervals were threaded into the cannula. This was then withdrawn leaving the microthermocouples visible lying under the serosa. The laser fibre was placed as for PDT directly on the bowel mucosa as near as possible to the thermocouples. The laser was set to deliver 100 mW for 500 sec. The temperature from each thermocouple was recorded continuously on tape for later computer analysis.

*Bursting strength of colon*

The bursting strength of the colon was measured by gaseous distention (Hawley, 1970). Segments of colon treated by PDT or hyperthermia and untreated control segments of similar lengths (4–5 cm) were slowly distended with a mixture of oxygen (95%) and carbon dioxide (5%) at a constant rate of 60 ml min$^{-1}$. The colon was placed in a water bath to detect the moment of air leakage. The pressure was measured by an in-line pressure transducer connected to an oscillograph (M10-120A, Micro Movements Ltd). This had been previously calibrated against a mercury manometer. The pressure was measured in mm Hg. The PDT lesions tested were produced by treating colon with 100 mW for 500 sec (50 joules) 1 h after sensitisation with 5 mg kg$^{-1}$ AlSPc. The bursting pressure was measured 1 h to 2 weeks after treatment. The colon was observed during and after bursting to see if it had ruptured at the site of the PDT lesion. Colons from unsensitised animals were treated with the same energy as for PDT (50 joules) but delivered at the hyperthermic laser power of 500 mW for 100 sec. The bursting strength of the thermally damaged colon was measured 3 h to 2 weeks after treatment.

*Light distribution in the colon*

The measurement of light distribution in living colonic wall proved difficult. When the light detecting fibre was inserted subserosally in a similar manner to that used for the thermocouples, colonic peristalsis and abdominal respiratory movements caused small alterations in its relationship to the emitting laser fibre. These movements were not critical for an array of 6 thermocouples but assumed greater importance for a single detecting fibre with a limited acceptance angle. In order to overcome these difficulties and obtain reproducible results, we had to devise an alternative method. Through a lateral abdominal incision the colon was mobilised. It was then opened on the antimesenteric border without disturbance to the blood supply. It was held loosely open by gently pinning the edge to a cork board. The laser fibre was placed in a modified clamp perpendicularly underneath the colon. It was directed to point upwards and placed just touching the mucosa without distortion of the colonic wall. The 400 micron detecting fibre was held perpendicularly in a micromanipulator on the opposite side of the colonic wall so that it was pointing directly downwards and just touching the colonic serosa. This procedure was performed under an operating microscope to position both emitting and receiving fibres correctly. The laser was switched on at 100 mW of power. The detecting fibre was connected to a photodiode and digital light meter and the fibre was moved along the serosal surface by 1 mm intervals using the micromanipulator and the intensity measured at each point. It remained just touching the serosa. The measurements were performed on colon 1 h after injection of 5 mg kg$^{-1}$ of AlSPc. The fall in the relative light intensity (fluence) was measured in arbitrary units as the distance from the emitting fibre increased. All intensity measurements were normalised to the measurement taken with the emitting and detecting fibres in direct apposition.

### Results

*Distribution of AlSPc in the colon*

The concentration of AlSPc extracted from colon as a function of time from i.v. injection of 5 mg kg$^{-1}$ is shown in Figure 1. The peak tissue concentration occurred at 1 h and then fell to a plateau between 3 h and 96 h. There was no AlSPc detectable 3 weeks after i.v. injection.

The fluorescence emission in arbitrary units (measured at fluorescence microscopy) from the colonic mucosa was 864±202 and from muscle was 144±51 measured 1 h after i.v. injection of 5 mg kg$^{-1}$ AlSPc. The measurements were corrected for any background fluorescence. There was therefore a sixfold difference between colonic muscle and mucosa.

**Figure 1** Concentration of extractable AlSPc in normal colon following i.v. injection of 5 mg kg$^{-1}$. Each point represents the mean (±s.d.) of 5 animals.

*Photodynamic damage to colon*

The area of PDT damage seen 72 h after phototherapy fell with the fall in tissue concentration of AlSPc 1 h to 1 month after i.v. injection of AlSPc (Figure 2). There was a large area of damage at 1 h which fell to a plateau between 3 and 96 h corresponding to the plateau in tissue concentration and falling again so that no damage was detectable at 3 weeks. However, the largest area of damage occurred when

**Figure 2** The mean area of PDT damage (100 mW for 500 sec, 50 J) as a function of the time from sensitisation (AlSPc 5 mg kg$^{-1}$) to light exposure. Each point represents the mean (±s.d.) from at least 4 animals.

treatment was immediately after i.v. injection. An intense vascular spasm was observed in the area treated. The first 5 animals treated in this manner died after 24 h and post-mortem examination showed the entire small bowel necrotic due to mesenteric ischaemia. This was due to light transmitted through the colon falling on the mesenteric vessels and causing an intense vascular spasm. In subsequent experiments the small bowel and mesentery were shielded and the lesion was restricted to the colon only.

Figure 3 shows the variation in necrosis with the dose of AlSPc and Figure 4 with applied energy (fluence). There was no difference between the lesions identifiable under the operating microscope in the control unsensitised animals and those that occurred when AlSPc was injected at $0.2\,mg\,kg^{-1}$ (energy 50 J) nor at 1 J of energy (dose $5\,mg\,kg^{-1}$).

**Figure 3** Mean radius of PDT damage (100 mW for 500 sec, 50 J), 1 h after sensitisation as a function of the administered dose of AlSPc (○). At the injected dose of $0.2\,mg\,kg^{-1}$ the radius of necrosis was no different from that in control unsensitised animals (●).

**Figure 4** Mean radius of PDT damage in the normal colon as a function of the applied energy for a laser power of 100 mW, 1 h after sensitisation with $5\,mg\,kg^{-1}$ AlSPc (○). 1 Joule of energy produced a lesion no different from that of control unsensitised animals (●). Each point represents the mean (±s.d.) from at least 3 animals.

### *Histology*

The histological progression of the damage was similar whether treatment with 100 mW of laser power for 500 sec was at 1 h or 48 h after sensitisation with $5\,mg\,kg^{-1}$. The initial events were vascular; 3 h after treatment there was acute vascular dilatation with evidence of vascular endothelial damage and leucocyte margination. There was some early cell death with a scattering of cells having pyknotic nuclei. By 12–24 h there was a lot of mucosal necrosis with inflammatory cell infiltrate; the muscle was oedematous and damaged but not dead. The endothelial wall of the blood vessels was dead with fibrinoid necrosis. By 48–96 h there was full thickness necrosis with inflammatory cell infiltrate. At the margins of the lesion the mucosa and serosa were necrotic but the muscle layer in between was viable having proved relatively resistant (Figure 5). Florid granulation tissue and adhesions were evident by 1 week and there was early regeneration of muscle and mucosa. Healing by regeneration was complete by 2–3 weeks. If there was any area of thermal damage this healed by fibrosis and scarring (Figure 6). Van Gieson and elastic van Gieson stains were performed to show the collagen architecture in the colon wall. In PDT lesions the fibrillar architecture of the submucosal collagen was maintained and appeared no different from normal colonic collagen. Hyperthermic damage produced clumped areas of collagen with destruction of the architecture (Figure 7).

### *Temperature*

The temperature measured in the sensitised colonic wall was 30–31°C. During treatment the maximum temperature rise adjacent to the laser fibre was 2°C. The colonic wall adjacent to the fibre never reached a hyperthermic temperature (>41°C) where thermal cellular destruction may become evident (Kinsey *et al.*, 1983; Dickson & Calderwood. 1980).

### *Bursting pressure*

The mean bursting pressure of untreated colon was $108 \pm 11$ mm Hg. There was no reduction in bursting pressure at any time after PDT despite full thickness damage (Figure 8). Bursting through the PDT lesion only occurred on 1 out of 35 occasions and that was not at a reduced pressure. Colon damaged thermally using 50 joules but delivered at a power of 500 mW for 100 sec showed reduction in the bursting pressure with bursting invariably occurring through the laser lesion. The bursting pressure returned to normal by 2 weeks. In addition immediate perforation occured during treatment in 20%.

### *Light distribution*

Figure 9 shows the fall in relative light intensity (fluence rate) as the distance from the laser fibre increases.

### Discussion

The aims of this investigation were to establish, the concentration of AlSPc in the normal colon as a function of time after injection, the distribution of AlSPc within the different layers of the colon, the factors controlling the extent of PDT damage in normal colon and finally to study the process of damage and repair.

In this study we have been careful to carry out control experiments in unsensitised animals to assess when hyperthermic effects occur. Using the method described we have only been able to see occasional microscopic thermal damage at the very tip of the fibre. We have not been able to detect a rise in temperature in the adjacent colon sufficient to cause hyperthermic cell death. The area of photodynamic damage increases as the total energy (fluence) increases. The energy in joules (log scale) was plotted against the radius of damage (Figure 4). The geometry of the rat colon is complex from an optical point of view. It was obvious at the time of treatment that light was spreading along the colon wall and not just being transmitted straight through, so scattering must play an important role in the transmission of light. It would be reasonable to expect an optical penetration depth of 1.5–3 mm and reflection coefficient (true reflection and backscattering) in the red of the order of 0.2–0.4 (L.O.Svaasand pers. comm.). Since the colonic wall thickness is 0.6 mm and is presumably larger than the mean free path between scattering events and smaller than the penetration depth, it is reasonable to expect that reflected light might be almost equal to light scattered

**Figure 5** Section of normal colon 72 h after PDT with 100 mW for 500 sec (50 J). Sensitisation with 5 mg kg$^{-1}$ AlSPc 1 h prior to light exposure. This section shows the margin of the lesion with full thickness damage on the left. On the right at the edges of the PDT damaged area there is complete mucosal necrosis but the muscle layer is intact and viable. (H&E × 100.)

**Figure 6** Section of normal colon 2 weeks after PDT with 100 mW for 500 sec (50 J). Sensitisation with 5 mg kg$^{-1}$ AlSPc 1 h prior to light exposure. There is complete regeneration except for the small central scar which measures just over 200 microns and is the area of thermal damage caused by direct contact with the tip of the 200 micron laser fibre. This serves to identify the area of treatment that has completely regenerated macroscopically. (H&E × 100.)

**Figure 7** Sections to show the submucosal collagen. *Upper left*: collagen architecture of normal untreated colon. *Upper right*: colonic collagen 72 h after PDT (100 mW for 500 sec, 50 J, sensitisation with 5 mg kg$^{-1}$ AlSPc 1 h prior to light exposure). Fibrillar elements of collagen are seen in the normal colon and remain present after PDT. *Lower centre*: destruction of the collagen architecture with dark staining of the collagen in unsensitised colon 72 h after hyperthermic treatment (500 mW for 100 sec, 50 J). Elastic van Gieson, ( x 400).

**Figure 8** Mean bursting pressure after photodynamic therapy (○) and hyperthermic treatment (▼) as a function of the time after treatment. Photodynamic therapy: 100 mW of laser power for 500 sec (50 J), 1 h after i.v. injection of 5 mg kg$^{-1}$ AlSPc. Hyperthermic treatment: 500 mW for 100 sec (50 J) in unsensitised animals. The mean bursting pressure of 15 normal rat colons is shown as the continuous black line (108 ± 11 mm Hg). Each point represents the mean (± s.d.) of at least 3 animals.

into the wall. Therefore backscattered light onto the opposite wall of the colon may be important and it is not surprising that the relationship between the radius of the necrotic zone and the delivered light energy is not simple. However, even if the geometry is difficult to analyse, it is clear that the diameter of PDT damage increases with the applied energy. Despite the difficulty in measuring light distribution in the colon, this measurement was important to quantify the effect of increasing the energy. We measured the radius of necrosis in the long axis of the colon at the different delivered energies (laser power(fluence rate) = 100 mW.) and calculated the light intensity at that radius by reference to the light distribution graph (Figure 9). The intensity at the edge of the necrotic zone multiplied by the length of exposure used should be constant if there is a threshold energy fluence for tissue necrosis. This is supported by the data in Table I which show that the energy fluence at threshold only varies by ~30% while the light intensity varies by 3000%. This result has important implications as it means that if the light intensity (fluence rate) is measured at any position, the time required to produce necrosis at that point can be predicted.

At the time of peak tissue concentration colonic muscle contains considerably less AlSPc than the mucosa. This differential uptake by the mucosa may be related to its

**Figure 9** The relative light intensity in normal colon sensitised with 5 mg kg$^{-1}$ AlSPc as a function of the distance from the emitting laser fibre (100 mW). Each point represents the mean of four animals with standard deviations. Light intensity was measured in arbitrary units after normalisation of the measurements to those taken with the detecting and emitting fibres in direct apposition.

**Table I** Energy fluence at the limit of the necrotic zone for variable light intensities and exposure times

| *Longitudinal radius of necrosis (mm)* | *Light intensity at margin of necrotic area. (arbitrary units)* | *Length of light exposure (sec)* | *Intensity X length light exposure (= energy fluence)* |
|---|---|---|---|
| 2.4 | 0.25 | 50 | 12.5 |
| 4.0 | 0.071 | 250 | 18 |
| 5.0 | 0.032 | 500 | 16 |
| 7.6 | 0.01 | 1000 | 10 |
| 8.3 | 0.0074 | 2000 | 15 |
| | Mean energy fluence = 14.3 ± 3 | | |

cellularity, rapid turnover and vascularity. The reduced uptake of AlSPc by the muscle explains why it remains relatively undamaged in areas of low light intensity at the margins of PDT lesions.

The area of necrosis for a given light dose varies with the level of extractable AlSPc. The plateau in Figure 2 mirrors the plateau in the extractable AlSPc concentration seen in Figure 1. There is no prolonged photosensitisation as has been found in the liver (Bown *et al.*, 1986). By 3 weeks after sensitisation no damage was produced with 50 J of light. The exception was when treatment was immediately after the i.v. injection of AlSPc. The high plasma levels produced profound vascular effects. The inadvertent irradiation of small bowel and mesentery at this time caused ischaemia and death.

For treatment at a fixed time after sensitisation, the total i.v. dose also influences the area of damage. Above 0.2 mg kg$^{-1}$ the size of the lesion increases with the logarithm of the dose of injected AlSPc.

It is surprising that few studies have been directed to the response of normal tissue to PDT. There is no major difference in the response of normal and neoplastic cells in culture (Moan *et al.*, 1981). The possibility of selective treatment occurs because of the retention of the sensitiser in malignant tissue relative to normal. Also laser light can be directed via flexible fibres to illuminate tumours selectively. Some studies have suggested that HpD is retained not in individual malignant cells but in the vascular stroma of malignant tumours (Bugelski *et al.*, 1981). An elegant study at Roswell Park showed that when tumour cells were transplanted to tissue culture immediately after phototherapy they grew normally, whereas those transplanted 12 h later were not viable. Vascular shut-down in the tumour was implicated (Henderson *et al.*, 1985). The vascular effects occurring with HpD phototherapy have been observed directly on tumours in sandwich chambers (Star *et al.*, 1986), and by measuring the fall in blood flow to the bladder and the jejunum after PDT (Selman *et al.*, 1985*a*, *b*). In our study the vascular effects were evident histologically 3 h after light exposure both 1 h and 48 h after AlSPc injection. They preceded the full thickness damage and consisted of vascular dilatation, red cell agglutination and endothelial cell damage. Endothelial cell damage has been implicated in HpD phototherapy (Berenbaum *et al.*, 1986). Mucosal necrosis was not evident until 12–24 h and full thickness destruction until 72 h. The colon heals by regeneration which is complete 2–3 weeks after treatment. Thermally damaged bowel heals leaving a fibrous scar. Tiny scars caused by very localised thermal damage in the area where the fibre touched the mucosa (0.2 mm in diameter) have proved useful for establishing the exact site of PDT damage 2 weeks after treatment when the macroscopic and microscopic appearance is essentially normal except for occasional patches of florid regeneration. Thermally induced fibrosis has occasionally resulted in fibrous strictures following Nd-YAG laser treatment of oesophageal and rectal tumours (Bown, 1986). These results suggest that scarring does not occur when PDT necrosis heals, which could prove useful in clinical applications.

An unexpected finding was that at no time after treatment was there a fall in the bursting pressure. A PDT lesion in which there was full thickness necrosis maintained its strength. It was unusual for the colon to burst through the laser lesion and when this occurred there was no reduction in bursting pressure. Hyperthermic treatment of colon produced a considerable reduction in bursting pressure and colonic perforation. No perforations were produced by PDT. Histological stains for collagen showed that the collagen architecture was spared during PDT. Hyperthermia causes alteration in this architecture.

AlSPc is an effective sensitiser for colonic PDT. The lesions heal by regeneration with no reduction in bursting pressure. Photodynamic therapy with AlSPc appears to be collagen sparing. The potential for colonic PDT with AlSPc seems to be important since it may offer a treatment for polyps and cancers without disturbing the collagen architecture and the strength of the colon so perforation would be unlikely. Also, as it is possible to induce fibrosis using hyperthermic Nd-YAG laser therapy (Bown, 1986), a combination of the two techniques may be useful. The differential uptake of AlSPc by experimental colonic tumours requires further investigation to assess whether any true selectivity can be achieved in treating these lesions.

This work was carried out in the Department of Surgery at University College Hospital London. Mr H. Barr is supported by a

Surgical Research Training Fellowship from the Wellcome Trust, Dr A. MacRobert by a training fellowship from the Medical Research Council, and Ms C.J. Tralau and Dr S.G. Brown by the Imperial Cancer Research Fund. The help of Prof R. Cherry, Dr D. Capey, and Mr I. Morrison of the Department of Chemistry, University of Essex was invaluable for the fluorescence microscopy as was that of Dr C.M. Collins for the histology, Dr T. Mills of the Department of Medical Physics at University College Hospital for continuous technical help and Prof L.O. Svaasand for advice on the theoretical analysis. The laser was purchased with a grant from the Stanley Thomas Johnson Foundation. The assistance of Mr P.D. Coleridge-Smith and Mr R. Svensen is also gratefully acknowledged.

### References

BARR, H., TRALAU, C.J., BARTON, T. & 5 others (1986). Photodynamic therapy with phthalocyanine sensitisation in the rat colon. *Br. J. Surg.*, **73**, 1037. (*Abstract*).

BEN-HUR, E. & ROSENTHAL, I. (1986). Photosensitisation of Chinese hamster cells by water-soluble phthalocyanines. *Photochem. Photobiol.*, **43**, 15.

BERENBAUM, M.C., HALL, G.W. & HOYES, A.D. (1986). Cerebral photosensitisation by haematoporphyrin derivative. Evidence for an endothelial site of action. *Br. J. Cancer*, **53**, 81.

BOWN, S.G., TRALAU, C.J., COLERIDGE-SMITH, P.D., AKDEMIR, D. & WEIMAN, T.J. (1986). Photodynamic therapy with porphyrin and phthalocyanine sensitisation: Quantitative studies in normal rat liver. *Br. J. Cancer*, **54**, 43.

BOWN, S.G. (1986). Endoscopic laser therapy for oesophageal cancer. *Endoscopy*, **18**, 26.

BUGELSKI, P.J., PORTER, C.W. & DOUGHERTY, T.J. (1981). Autoradiographic distribution of hematoporphyrin derivative in normal and tumor tissue of the mouse. *Cancer Res.*, **41**, 4606.

CHAN, W.S., SVENSEN, R., PHILIPS, D. & HART, I.R. (1986). Cell uptake, distribution and response to light of aluminium chloro sulphonated phthalocyanine, a potential anti-tumour photosensitiser. *Br. J. Cancer*, **53**, 255.

DARWENT, J.R., McCUBBIN, I. & PHILIPS, D. (1982). Excited singlet and triplet state electron transfer reactions of aluminium sulphonated (III) Phthalocyanine. *J. Chem. Soc. Faraday Trans.*, **2**, 78, 347.

DICKSON, J.A. & CALDERWOOD, S.K. (1980). Temperature range and selective sensitivity of tumors to hyperthermia: A critical review. *Ann. N.Y. Acad. Sci.*, **335**, 180.

DOIRON, D.R. & GOMER, C.J. (eds.) (1984). *Porphyrin Localisation and Treatment of Tumors*. Alan R. Liss: New York.

DOUGHERTY, T.J., POTTER, W.R. & WEISHAUPT, K.R. (1984). The structure of the active component of hematoporphyrin derivative. In *Porphyrin Localisation and Treatment of Tumors*, Doiron & Gomer, (eds.) p. 301. Alan R. Liss: New York.

HAWLEY, P.R. (1970). *The Aetiology of Colonic Anastomotic Leaks with Specific Reference to the Role of Collagenase*. M.S. Thesis, London University.

HENDERSON, B.W., WALDOW, S.M., MANG, T.S., POTTER, W.R. MALONE, P.B. & DOUGHERTY, T.J. (1985). Tumor destruction and kinetics of tumor cell death in two experimental mouse tumors following photodynamic therapy. *Cancer Res.*, **45**, 572.

KESSEL, D. (1985). Proposed structure of the tumor localising component of 'hematoporphyrin derivative'. In *Photodynamic Therapy of Tumours and Other Diseases*. Jori and Perria (eds.) p. 1. Libreria Progett, Padova.

KINSEY, J.H., CORTESE, D.A. & NEEL, H.B. (1983). Thermal considerations in murine tumor killing using hematoporphyrin derivative phototherapy. *Cancer Res.*, **43**, 1562.

McCUBBIN, I. (1985). *Photochemistry of Some Water Soluble Phthalocyanines*. PhD. Thesis. University of London.

MOAN, J., STEEN, H.B., FEREN, K. & CHRISTENSEN, T. (1981). Uptake of Haematoporphyrin derivative and sensitized photoinactivation of C3H with different oncogenic potential. *Cancer Lett.*, **14**, 291.

MOAN, J. (1986). Porphyrin Photosensitisation and phototherapy. *Photochem. Photobiol.*, **43**, 681.

NELSON, J.S., WRIGHT, W.H. & BERNS, M.W. (1985). Histopathological comparison of the effects of hematoporphyrin derivative on two different murine tumors using computer-enhanced digital video. *Cancer Res.*, **45**, 5781.

ROSENTHAL, I., MURALI KRISHNA, C., RIESZ, P. & BEN-HUR, E. (1986). The role of molecular oxygen in the photodynamic effect of phthalocyanines. *Radiation Res.*, **107**, 136.

SELMAN, S.H., MILLIGAN, A.J., KREIMER-BIRNBAUM, M., KECH, R.W., GOLDBLATT, P.J. & BRITTON, S.L. (1985*a*). Haematoporphyrin derivative photochemotherapy of experimental bladder tumours. *J. Urol.*, **133**, 330.

SELMAN, S.H., KREIMER-BIRNBAUM, M., GOLDBLATT, P.J., ANDERSON, T.S., KECK, R.W. & BRITTON, S.L. (1985*b*). Jejunal blood flow after exposure to light in rats injected with hematoporphyrin derivative. *Cancer Res.*, **45**, 6425.

SPIKES, J.D. (1986). Phthalocyanines as photosensitisers in biological systems and for the photodynamic therapy of tumors. *Photochem. Photobiol.*, **43**, 691.

STAR, W.M., MARIJNISSEN, H.P.A., VAN DER BERG-BLOK, A.E., VERSTEEG, J.A.C., FRANKEN, K.A.P. & RHEINHOLD, H.S. (1986). Destruction of rat mammary tumor and normal microvasculature by hematoporphyrin derivative photoradiation observed *in vivo* in sandwich observation chambers. *Cancer Res.*, **46**, 2532.

TRALAU, C.J., MACROBERT, A.J., COLERIDGE-SMITH, P.D., BARR, H. & BOWN, S.G. (1987). Photodynamic therapy with phthalocyanine sensitisation: Quantitative studies in a transplantable fibrosarcoma of rats. *Br. J. Cancer*, **55**, 389.

WEISHAUPT, K.R., GOMER, C.J. & DOUGHERTY, T.J. (1976). Identification of singlet oxygen as the cytotoxic agent in photoinactivation of a murine tumor. *Cancer Res.*, **36**, 2326.